# Teleportation of a Toffoli gate among distant solid-state qubits with quantum dots embedded in optical microcavities

**DOI:** 10.1038/srep11321

**Published:** 2015-07-30

**Authors:** Shi Hu, Wen-Xue Cui, Dong-Yang Wang, Cheng-Hua Bai, Qi Guo, Hong-Fu Wang, Ai-Dong Zhu, Shou Zhang

**Affiliations:** 1 Department of Physics, College of Science, Yanbian University, Yanji, Jilin 133002, China; 2 Department of Physics, Harbin Institute of Technology, Harbin 150001, China

## Abstract

Teleportation of unitary operations can be viewed as a quantum remote control. The remote realization of robust multiqubit logic gates among distant long-lived qubit registers is a key challenge for quantum computation and quantum information processing. Here we propose a simple and deterministic scheme for teleportation of a Toffoli gate among three spatially separated electron spin qubits in optical microcavities by using local linear optical operations, an auxiliary electron spin, two circularly-polarized entangled photon pairs, photon measurements, and classical communication. We assess the feasibility of the scheme and show that the scheme can be achieved with high average fidelity under the current technology. The scheme opens promising perspectives for constructing long-distance quantum communication and quantum computation networks with solid-state qubits.

Quantum computation, which promises to speed up the solution of a number of mathematical tasks, has attracted tremendous interests. Quantum logic gates are fundamental elements of quantum computation. It is well known that single-qubit unitary gates together with two-qubit controlled-NOT (CNOT) gates are sufficient for construction of universal quantum computation network in principle. Unfortunately, it is too complex to implement most algorithms if only single- and two-qubit gates are available with the increase of the number of qubits. Therefore, it is significant to seek methods to implement multiqubit gates directly, which is generally believed to provide a simpler design, a faster operation, and a lower decoherence. As a universal three-qubit logic gate, Toffoli gate that is widely used in phase estimation^1^, complex quantum algorithms^2^, quantum error correction^3^, and fault tolerant quantum circuits^4^ plays a key role in forming a universal quantum computation architecture^5^,^6^. Many schemes^7^,^8^,^9^,^10^ have been proposed to implement Toffoli gate among local nodes. To realize distributed quantum computation, on the other hand, collective quantum gate operations often need to be implemented on the qubits at distant nodes. It is thus useful and interesting to investigate the local implementation of nonlocal quantum gates between spatially separated nodes. Teleportation of quantum gates, which is a quantum remote control for the optimal realization of nonlocal quantum operations by taking advantage of local operations, classical communication, and prior shared entanglement (that is, a collective quantum gate operation acting on the local qubits is teleported and acts on an arbitrary states belonging to remote qubits without physically sending the device), is a essential way of realizing quantum information processing networking and teleportation-based building blocks of quantum computation and quantum communication. In Refs. [11, 12, 13, 14] the general idea for the teleportation of quantum gates has been discussed in detail, and it has been found that two shared ebits (maximally entangled pairs of qubits) and four cbits (bits of classical communication) are necessary and sufficient for the implementation of a nonlocal Toffoli gate. Recently, a series of works on the physical realization of teleportation of quantum gate operations on one and two qubits have been proposed and made some interesting progress both in theory and in experiment^15^,^16^,^17^,^18^,^19^,^20^. From our point of view, therefore, it is very important and useful to investigate how to extend the teleportation of quantum gate operations to the cases of multiqubits.

Over the past decade, many physical systems have been studied in order to realize quantum computation, such as cavity quantum electrodynamics (QED) systems^21^,^22^,^23^,^24^,^25^, ion trap systems^26^,^27^, nuclear magnetic resonance (NMR) systems^28^, linear optics systems^29^, superconducting circuit systems^30^,^31^, and so on. Recently, semiconductor quantum dots (QDs) have been widely studied as promising candidates for solid-state-based quantum information processing and quantum computation. Bonato *et al.*^32^ reported an important work that introduced an interface between the polarization degree of freedom of a photon and the spin of an electron confined in a quantum dot embedded in a microcavity operating in the weak-coupling regime. The interface is based on spin selective photon reflection from the cavity and can be used to construct a CNOT gate, a multiphoton entangler and a photonic Bell-state analyzer. Hu and Rarity^33^ proposed efficient loss-resistant schemes for heralded state teleportation and entanglement swapping using a single quantum-dot spin in an optical microcavity based on giant circular birefringence. Based on such quantum-dot-microcavity coupled system, furthermore, some other attractive schemes have also been proposed to realize quantum computation and quantum information processing^34^,^35^,^36^,^37^,^38^,^39^,^40^.

In this paper, inspired by the above works, we propose an efficient scheme to teleport a Toffoli gate among three distant electron spins with quantum dots in optical microcavities, resorting to local linear optical operations, an auxiliary electron spin, and two circularly-polarized entangled photon pairs. The Toffoli gate can be successfully teleported from acting on local qubits to acting on remote qubits in a deterministic way by performing measurements on the photons and the auxiliary electron spin. The proposed scheme may open promising possibilities for distributed quantum computation, long-distance quantum communication, and the construction of remote quantum-information-processing networks.

## Results

### Cavity-induced photon-polarization electron-spin interface

**Figure 1:**
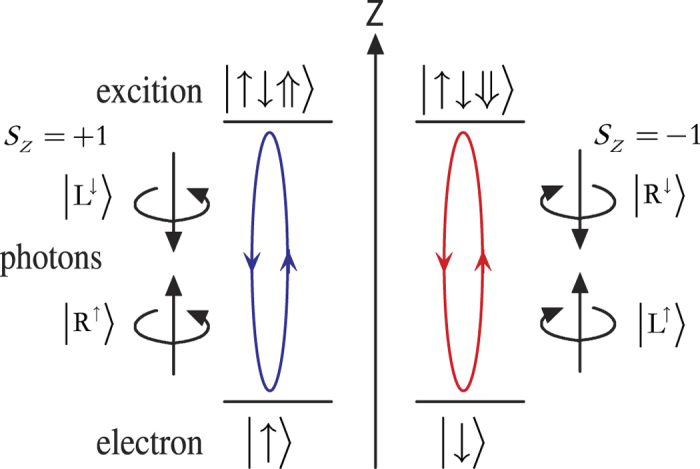
Relevant energy level and optical selection rules for the optical transition of *X*^−^. Here 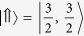 and 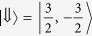 represent heavy hole states with spin 3/2 and −3/2 components and the superscript arrow indicate their propagation direction along the *z* axis. The quantization axis is the *z* axis.

We consider a singly charged GaAs/InAs QD embedded in a double-sided optical microcavity with two partially reflective mirrors in the top and bottom. The four relevant energy levels and optical selection rules is shown in Fig. 1. The optical excitation of the system will produce an exciton (*X*^−^) with negative charges and the charged exciton consists of two electrons bound in one hole. There are two kinds of optical dipole transitions between the electron and the exciton *X*^−^, under Pauli’s exclusion principle. For a photon with *s*_*z*_ = −1 (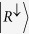 or 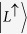), if the electron is in the state 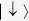 it feels a coupled (hot) cavity and will be reflected with both the polarization and propagation direction of the photon being flipped. While if the electron is in state 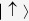, the photon feels an uncoupled (cold) cavity and will be transmitted by the cavity, acquiring a *π* phase shift relative to the reflected photon. Likewise, for the photon with *s*_*z*_ = +1 (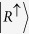 or 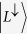), if the electron is in the state 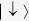, it will transmit the cavity. If the electron is in the state 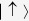 the photon is reflected by the optical cavity. That is, the electron-spin-cavity system behaves like a beam splitter. According to the above discussion the dynamics of the interaction between photon and electron in QD-microcavity coupled system is described as below^32^


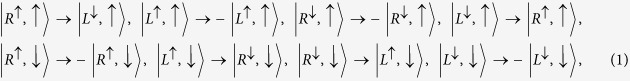


where 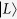 and 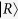 represent the states of the left- and right-circularly-polarized photons, respectively. The superscript arrow in the photon state indicates the propagation direction along the *z* axis, 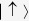 and 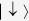 denote the direction of the electron spins.

### Teleportation of a Toffoli gate among distant solid-state qubits

**Figure 2:**
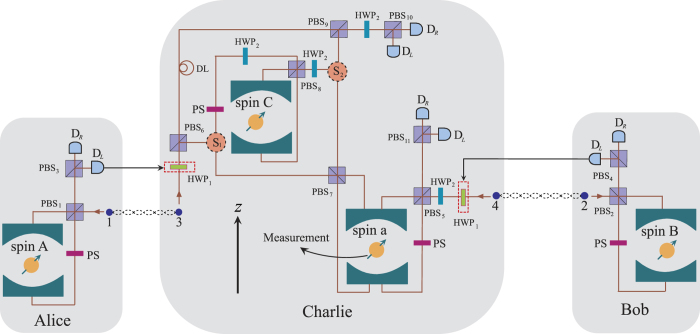
Schematic of teleportation of a Toffoli gate for spin qubits using quantum-dot-microcavity coupled systems. Here PBS_*i*_ denote polarizing beam splitters in the circular basis, HWP_1_ and HWP_2_ are half-wave plates, PS is phase shifter making |*R*〉 and |*L*〉 become −|*R*〉 and −|*L*〉, S_1_ and S_2_ are optical switches, D_*L*_ and D_*R*_ are non-photon-number-resolving detectors, and DL is the time-delay device making that the two wave-packs of photon 3 reach PBS_9_ simultaneously. The feedback operations in dashed box are performed or not depend on corresponding measurement results of photons 1 and 2.

We now show how to teleport a Toffoli gate among three spatially separated electron spin qubits using quantum-dot-microcavity coupled system based on the photon-spin interaction rules discussed above. The schematic is shown in Fig. 2. The Toffoli gate is among electron spins *A*, *B* and *C* in which electron spins *A* and *B* are control qubits and electron spin *C* is target qubit. In Fig. 2, photons 1 and 3 (2 and 4) are prepared in the state


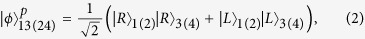


and electron spins *A*, *B*, and *C* are initialized to the following product state


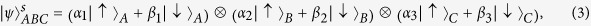


where *α*_*i*_ and *β*_*i*_ satisfy |*α*_*i*_|^2^ + |*β*_*i*_|^2^ = 1. Here photon 1 and electron spin *A* are in Alice’s site, photon 2 and electron spin *B* are in Bob’s site, and photons 3 and 4 and electron spin *C* are in Charlie’s site, respectively.

To realize the teleportation process, Charlie introduces an ancillary electron spin *a*, initialized in the state 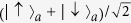. Firstly, let photons 1 and 2 pass through the PBS_1_ and PBS_2_, respectively. Here PBS is the polarizing beam splitter in the circular basis, which transmits the right circularly polarized photon 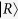 and reflects the left circularly polarized photon 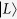. Then photons 1 and 2 pass through a phase shifter PS, respectively. The action of PS is to complete the transformation: 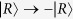 and 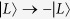. The state of the photons 1 and 3 (2 and 4) becomes


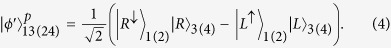


Then photons 1 and 2 enter into the optical microcavities interacting with electron spins *A* and *B*, respectively. After that, photons 1 and 2 exit from the optical microcavities and pass through the PBS_1_ and PBS_2_ again. Then the state of the system is given by


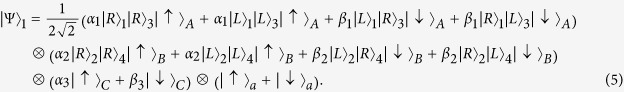


Secondly, Alice and Bob perform a measurement on photons 1 and 2, respectively. If Alice measures photon 1 in the state 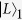, the photon 3 in Charlie’s site is rotated by a half-wave plate HWP_1_, whose action is given by the transformation: 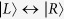; if the measurement result is 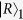, nothing is done on photon 3. While for the measurement results of Bob’s, if photon 2 is measured in state 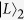, the photon 4 in Charlie’s site is rotated by HWP_1_; if the measurement result is |*R*〉_2_, nothing is done on photon 4. After photons 1 and 2 are detected, we obtain


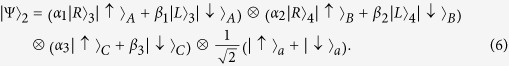


Thirdly, let photon 4 pass through HWP_2_, PBS_5_, and PS and then enter into the optical microcavities interacting with electron spin *a*. The action of HWP_2_ is to complete the transformation


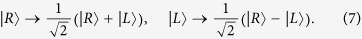


After the interaction between photon 4 and spin *a*, Charlie performs a Hadamard gate operation, which can be achieved by a *π*/2 microwave pulse^41^, on electron spin *a* to accomplish the transformation


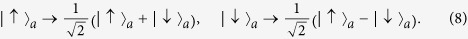


Then the state of the system becomes


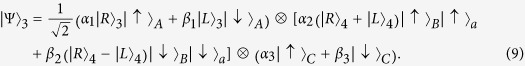


Fourthly, let photon 3 pass through PBS_6_. When the state of photon 3 is |*L*〉_3_, it will be reflected by PBS_6_ and pass through optical switch S_1_ and PBS_7_ successively, and then it interacts with electron spin *a*. When the state of photon 3 is |*R*〉_3_, it will be transmitted by PBS_6_ and dose not interact with electron spins *C* and *a* during the following process. That is, PBS_6_ splits photon 3 into two wave-packets. After the component |*L*〉_3_ of the photon 3 interacts with spin *a*, the state of the system is changed to


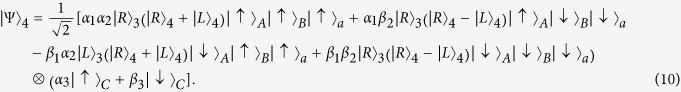


Fifthly, let the photon pass through S_2_, HWP_2_, and PBS_8_ and enter into the optical microcavity interacting with electron spin *C*. Before and after the interaction between the photon and electron spin *C*, a Hadamard gate operation is performed on electron spin *C*, respectively. When the photon leaves the optical microcavity, it passes through PBS_8_, HWP_2_, and PS, the state of the system becomes


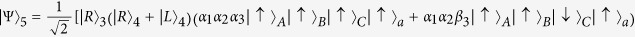



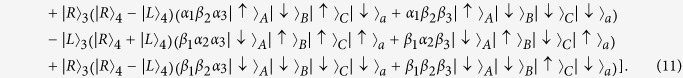


Sixthly, the photon passes through S_1_ and PBS_7_ successively and then it interacts with electron spin *a* again. After that, the photon passes through S_2_ and arrives at PBS_9_, and in the meantime another wave-packet of photon 3 arrives PBS_9_ too. Namely, the two wave-packets of photon 3 pass through PBS_9_ simultaneously. The state of the system is given by


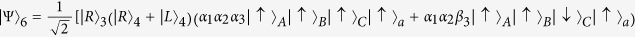



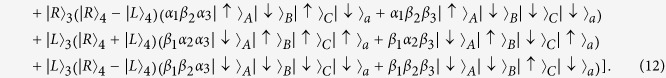


Seventhly, let photon 3 pass through HWP_2_ and a Hadamard gate operation is performed on electron spin *a*. Finally, Charlie performs the measurements on photon 3, photon 4, and electron spin *a*. The measurement of electron spin is discussed in detail in the methods section. After the measurement and appropriate single-qubit gate rotation on electron spins *A* and *B* (see Table 1), the final state of electron spins *A*, *B*, and *C* is written as


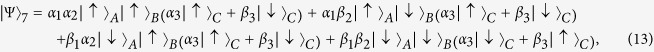


which achieves the deterministic teleportation of a Toffoli gate among three remote electron spins successfully.

## Discussion

We now begin to discuss the feasibility of the present scheme. The basic module in the present scheme is QD-cavity system, which determines the performance of the present scheme. In equation (1) we give the optical transition rules without regarding to the side leakage and cavity loss. In a realistic cavity, however, the factors are not negligible. In the weak excitation approximation the reflection and transmission coefficients of the cavity are described by^42^


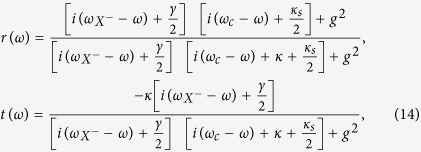


where *g* is the coupling strength between *X*^−^ and the cavity field, *κ*, *κ*_*s*_, and *γ* are the cavity field decay rate, leaky rate, and *X*^−^ dipole decay rate, respectively. *ω*, *ω*_*c*_, and 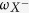 are the frequencies of the input photon, cavity mode, and the spin-dependent optical transition, respectively. The coefficients can be calculated from the Heisenberg equations of motion for the cavity field operator 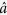 and *X*^−^ dipole operator *σ*_−_ in the interaction picture(see methods section). In our work we consider 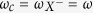, the reflection and transmission coefficients of the coupled cavity and uncoupled cavity (*g* = 0) are given by


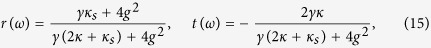


and


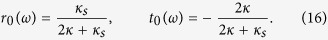


Therefore, the rules of optical transitions in a realistic cavity become^42^


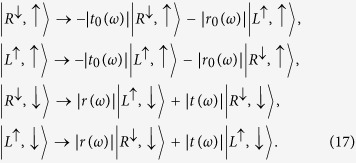


In order to quantitatively test the quality of the present scheme, we introduce the gate fidelity defined as^43^


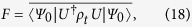


where the overline indicates average over all possible input states |Ψ_0_〉, *U* is the ideal three-qubit Toffoli gate, and *ρ*_*t*_ = |Ψ_*t*_〉〈Ψ_*t*_|, with |Ψ_*t*_〉 being the final state of the realistic Toffoli gate operation in the present scheme. Assume that the three electron spins are initially in the general state 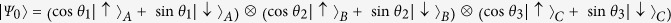 , the ideal target state is |Ψ_*s*_〉. Then the average fidelity of the Tofolli gate can be written as


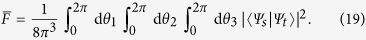


**Figure 3:**
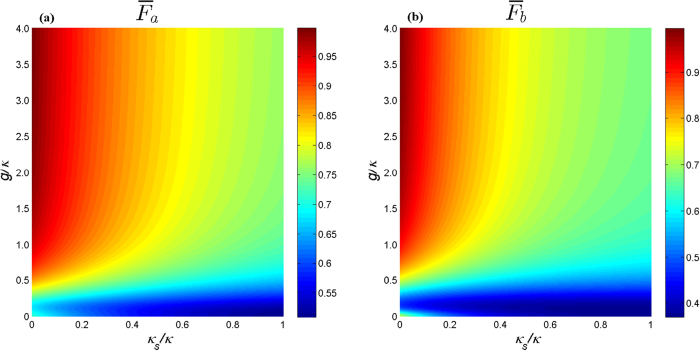
The average fidelity of the teleportation of Toffoli gate versus the normalized coupling strengths *κ*_*S*_/*κ* and *g*/*κ*. (**a**) The fidelity corresponding to that the measurement result of photon 3 is |*R*〉_3_. (**b**) The fidelity corresponding to that the measurement result of photon 3 is |*L*〉_3_ (see Table 1). Here we have set *γ* = 0.1*κ*.

In Fig. 3 we plot the fidelities with the side leakage *κ*_*s*_/*κ* and the coupling strength *g*/*κ* corresponding to different measurement results (see Table 1), showing that in the case of *κ*_*s*_ ≪ *κ* we can get a high average gate fidelity. Experimentally, the weak coupling with *g* < (*κ* + *κ*_*s*_)/4 can be easily achieved, while for the strong coupling with *g* > (*κ* + *κ*_*s*_)/4 has also been demonstrated in various microcavity- and nanocavity-QD systems recently^44^,^45^,^46^,^47^ and *g*/(*κ* + *κ*_*s*_) ≃ 0.5 and *g*/(*κ* + *κ*_*s*_) ≃ 2.4 have been reported^44^,^47^. In the present scheme, if setting *κ*_*s*_ = 0.01*κ*, *g* = 2.4*κ*, we can obtain 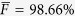; even when setting *κ*_*s*_ = 0.1*κ*, *g* = 0.27*κ*, we also can obtain 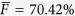. Therefore, the present scheme can work in both the weak coupling and the strong coupling regimes. On the other hand, the preparation of electron spin superpositions and its fast initialization and manipulation have been demonstrated in Refs. [41,48, 49, 50, 51, 52, 53, 54, 55]. The entangled pairs of photons can be produced by the well-known spontaneous parametric down-conversion^56^ and the photon detectors in our scheme are non-photon-number-resolving detectors, which greatly decreases the high-quality requirements of photon detectors in practical realization. Besides, some other small factors caused by spin decoherence and the trion dephasing also can reduce the gate fidelity, which has been discussed in detail in Ref. [33] and we thus dot not repeat here. Therefore, the present scheme is feasible with current technology.

In conclusion, we put forward an effective scheme to implement quantum remote control with quantum dots embedded in optical microcavities by using local linear optical operations, an auxiliary electron spin, two circularly-polarized entangled photon pairs, photon measurement, and classical communication. The scheme is achieved in a deterministic way by the sequential detection of photons and auxiliary electron spin and the single-qubit rotations of photon and electron spin in principle. The calculated results show that our scheme can work in both the weak coupling and the strong coupling regimes and when *κ*_*s*_ ≪ *κ* we can get a fidelity near unity in strong coupling regime. The scheme is feasible with current technology and would open promising perspectives for long-distance quantum communication, distributed quantum computation and remote quantum-information-processing.

## Methods

### Manipulation and measurement of the electron spin in QD

The QD-spin superposition state can be prepared by performing single spin-qubit rotations with picosecond optical pulses^57^,^58^. Ultrafast optical coherent manipulation of a QD-spin qubit has been demonstrated in a picosecond or femtosecond time scale^58^,^59^, and an ultrafast *π*/2 spin rotation can be used to complete a Hadamard operation on a spin qubit. The projection measurement of the spin under the basis {|↑〉,|↓〉} can be achieved with the help of an auxiliary circularly polarized photon. If a right-circularly polarized photon |*R*〉 is initially sent into the cavity along the *z* axis, after interacting with QD-cavity system, the joint state of the photon and electron becomes


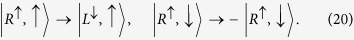


Obviously, the projection measurement of the electron spin can be completed by detecting the reflection and transmission of the photon. If the photon is detected on the reflection port, the electron spin is projected into the state |↑〉; if the photon is detected on the transmission port, the electron spin is projected into the state |↓〉.

### Input-output relation of QD double-sided cavity system

The reflection and transmission coefficients of the QD-cavity system can be calculated from the Heisenberg equations of motion for the cavity field operator 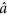 and *X*^−^ dipole operator *σ*_−_ in the interaction picture^60^


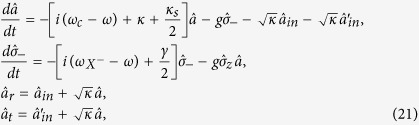


where 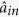, 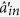 and 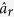, 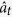 are the two input and the two output fields operators of the double-side cavity, respectively. And other parameters are the same as equation (14). The reflection and transmission coefficients in equation (14) can be obtained in the approximation of weak excitation where the charged QD is predominantly in the ground state with 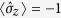.

## Additional Information

**How to cite this article**: Hu, S. *et al.* Teleportation of a Toffoli gate among distant solid-state qubits with quantum dots embedded in optical microcavities. *Sci. Rep.* **5**, 11321; doi: 10.1038/srep11321 (2015).

## Figures and Tables

**Table 1:** The correspondence to the measurement results of photon 3, photon 4, and electron spin *a*, the corresponding single-qubit operations on electron spins *A* and *B*, and the average fidelities for teleporting a Toffoli gate among distant three electron spins.

**Measurement results**	**Operations**	**Fidelities**
|*R*〉_3_|*R*〉_4_|↑〉_*a*_	*I*^*A*^⊗*I*^*B*^	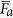
|*R*〉_3_|*L*〉_4_|↓〉_*a*_		
|*R*〉_3_|*R*〉_4_|↓〉_*a*_	*I*^*A*^⊗*σ*^*B*^	
|*R*〉_3_|*L*〉_4_|↑〉_*a*_		
|*L*〉_3_|*R*〉_4_|↑〉_*a*_	*σ*^*A*^⊗*I*^*B*^	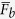
|*L*〉_3_|*L*〉_4_|↓〉_*a*_		
|*L*〉_3_|*R*〉_4_|↓〉_*a*_	*σ*^*A*^⊗*σ*^*B*^	
|*L*〉_3_|*L*〉_4_|↑〉_*a*_		
